# Risk Scores for Prediction of Major Cardiovascular Events in Non-Alcoholic Fatty Liver Disease: A No Man’s Land?

**DOI:** 10.3390/life13040857

**Published:** 2023-03-23

**Authors:** Liliana Gheorghe, Roxana Nemteanu, Andreea Clim, Gina Eosefina Botnariu, Irina Iuliana Costache, Alina Plesa

**Affiliations:** 1 Department of Radiology, “Grigore T. Popa” University of Medicine and Pharmacy, 700115 Iasi, Romania; 2 Radiology Clinic, “St. Spiridon” County Clinical Emergency Hospital, 700111 Iasi, Romania; 3 Medical I Department, “Grigore T. Popa” University of Medicine and Pharmacy, 700115 Iasi, Romania; andreea.clim13@yahoo.com (A.C.); alinaplesaro@yahoo.com (A.P.); 4 Institute of Gastroenterology and Hepatology, Saint Spiridon Hospital, 700111 Iasi, Romania; 5 Diabetes, Nutrition and Metabolic Diseases Department, University of Medicine and Pharmacy “Gr. T. Popa”, 700115 Iasi, Romania; 6 Cardiology Clinic, “St. Spiridon” County Clinical Emergency Hospital, 700111 Iasi, Romania

**Keywords:** cardiovascular risk, non-alcoholic fatty liver disease, biopsy-free risk scores

## Abstract

Over the past 100 years, cardiovascular disease (CVD) has become a leading cause of mortality and morbidity in developed countries, and similar trends have occurred for chronic liver disease. Subsequent research also indicated that people with non-alcoholic fatty liver disease (NAFLD) had a twofold increased risk of CV events and that this risk was doubled in those with liver fibrosis. However, no validated CVD risk score specific for NAFLD patients has yet been validated, as traditional risk scores tend to underestimate the CV risk in NAFLD patients. From a practical perspective, identifying NAFLD patients and assessing severity of liver fibrosis when concurrent atherosclerotic risk factors are already established may serve as an important criterion in new CV risk scores. The current review aims to assess current risk scores and their utility for the prediction of CV events among patients with NAFLD.

## 1. General Considerations and Epidemiological Trends

Obesity is a multifactorial relapsing disease, as defined by the International Classification of Disease [1]. According to the recent World Health Organization (WHO) reports, it is estimated that about 2 billion adults are overweight, whilst 650 million are obese worldwide [2]. By 2030 it is predicted that one in five women and one in seven men will be living with obesity, resulting in over 1 billion people globally [3,4]. Addressing obesity is critical because it is associated with a range of diseases including type 2 diabetes (T2DM), chronic and acute heart and liver disease, and even malignancy [5]. Obesity, like all chronic diseases, has a wide range of drivers and determinants. The causes of obesity vary from heredity to biology, healthcare accessibility, mental health, processed food intake, and commercial and environmental factors [4,5]. These risk factors interact and compound one another across several systems, resulting in the trends we see today. In addition, obesity is a relapsing disease, which means that without addressing obesogenic environments, individuals remain exposed to the same triggering factors and predictable outcomes [6]. 

Being overweight or obese is among the main risk factors for developing non-alcoholic fatty liver disease (NAFLD), which is considered to be the most prevalent chronic liver disease worldwide [7]. NAFLD is the fastest growing obesity-related non-communicable disease and is a strong predictor of liver and cardiovascular mortality [8]. NAFLD is estimated to affect 32.4% of the world’s population and represents a spectrum of liver disease that ranges from simple steatosis to steatohepatitis (NASH), found in 30–70% of patients upon biopsy, with or without fibrosis [8,9]. 

However, a new concept is emerging that is known as metabolic-associated fatty liver disease (MAFLD), which is a heterogenous clinical entity. The diagnostic criteria of MAFLD requires the presence of metabolic risk factors in the setting of hepatic steatosis, includes individuals with other concomitant liver diseases, and excludes those with hepatic steatosis who do not fulfill the metabolic risk criteria [10]. Large cohort studies with longer follow-up periods reported that MAFLD patients had a higher risk of all-cause mortality and a higher risk of cardiovascular mortality compared to NAFLD patients who did not register an increase in the risk of all-cause mortality. However, advanced fibrosis in MAFLD was associated with higher estimates [11,12]. The conversion of terminology from NAFLD to MAFLD is not simply a change to a better-suited name but also a shift in the phenotypic characteristics of individuals who meet the criteria for MAFLD [12]. Recent papers reinforced the need to better categorize patients, and the change from NAFLD to MAFLD criteria may identify a greater number of individuals with metabolically complicated fatty liver and increased risk for CVD [10,11,12]. NAFLD is a part of a broader multi-system disease that also includes obesity, diabetes, high blood pressure, and high cholesterol. Therefore, redefining NAFLD as MAFLD may help improve our understanding of predictors that increase the risk of death, as reported by a number of papers [11,12].

The majority of people with NAFLD will not develop liver fibrosis or cirrhosis, yet some studies argue that by 2030, NASH will become the leading cause of liver transplant in the USA [13]. An analysis issued by the Global Burden of Disease Study in 2017 showed that liver cancer due to NASH displayed an alarming increase of 42.3% from 2007 and an overall increase of deaths from cancers by 25.4% between 2007 and 2017 [14]. Nevertheless, as obesity and metabolic syndrome are strongly associated with NAFLD, its prevalence will likely continue to grow substantially over the next decade [15]. The most important driver of NAFLD is dysfunctional visceral adipose tissue. NAFLD developing in the absence of visceral fat accumulation is rare and probably represents a distinct entity. Despite body mass index (BMI) being a poor indicator of visceral adiposity, and despite increased visceral adiposity frequently being observed as an underlying factor in people with NAFLD who are considered lean, people who are overweight or obese are at high risk of developing NAFLD compared with thin people [15,16].

As previously reported, chronic liver disease (CLD) and cardiovascular diseases (CVDs) are stealing the spotlight in current epidemiological trends concerning morbidity and mortality rates. CVDs, which include ischemic heart disease, acute myocardial infarction, and stroke, are the most common non-communicable diseases globally, responsible for an estimated 17.8 million deaths in 2017 [17]. At a global scale, total deaths from CVD increased by nearly 21% between 2007 and 2017 and were greater for men than for women at most ages [13,17]. As they progress asymptomatically and are usually diagnosed late when intervention is less effective, higher mortality rates are to be expected. Recently, it has become increasingly evident that NAFLD is a multisystem disease that affects many extra-hepatic organ systems, including the heart and vascular structures [18,19,20]. Therefore, the heart–liver axis is a new dynamic and complex soon-to-be-unraveled field of interest for the scientific community [19,20]. The paradigm is changing, and non-hepatologists should be attentive when assessing patients with CVD and potential liver involvement (and vice versa, as this association is no longer considered benign).

In this manuscript, we aimed to review current risk scores and their utility for the prediction of cardiovascular events among patients with NAFLD. In light of more recent discoveries, we performed a literature search between January 2012 and January 2023 on MEDLINE, EMBASE, Web of Science, Scopus, DDW.org, and ClinicalTrials.gov for English articles. The search terms used included, but were not limited to, “NAFLD”, “non-alcoholic fatty liver disease”, “risk scores”, “cardiovascular risk scores”, “cardiovascular risk factors”, “mortality prediction scores”, “cardiovascular risk assessment”, and “prediction models”. The cited articles were selected based on their relevance to the review objective.

## 2. Proposed Pathogenic Mechanisms for the Occurrence of CVD in NAFLD Patients

**Figure 1:**
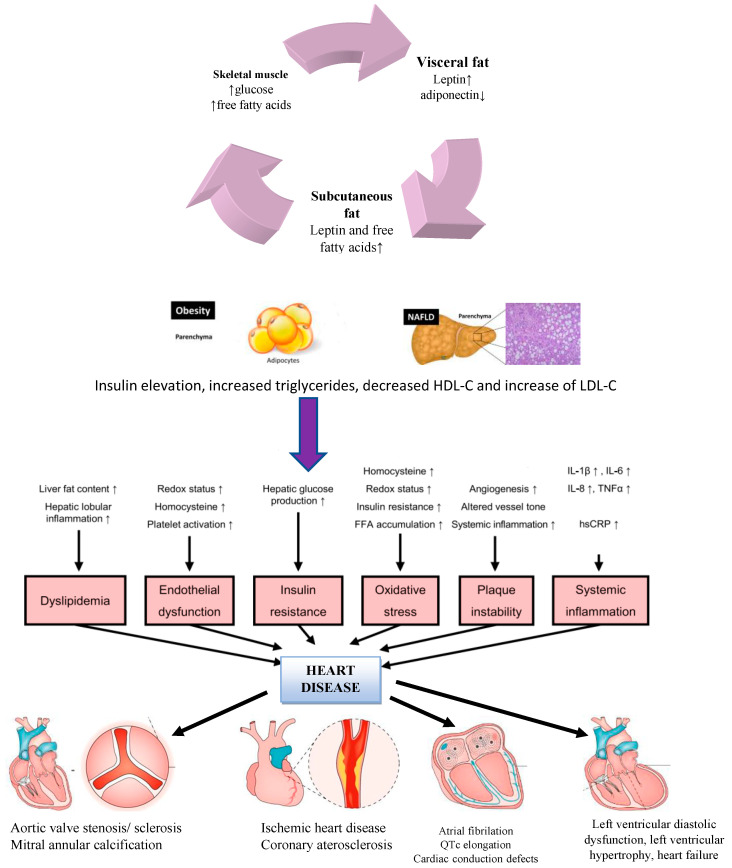
Complementary pathophysiological mechanisms involving NAFLD and CVD.

Whether liver disease in NAFLD confers any additional CV risk, or whether an increase in CV risk in NAFLD is due to associated CVD risk factors, is still a matter of avid discussion. Understanding whether the liver disease in NAFLD contributes to additional CVD risk is important, as it is plausible that treatment of the liver disease may reduce the overall risk of CVD above the treatment of NAFLD-associated risk factors [21]. Recent papers have reported interesting aspects concerning the link between NAFLD and CVD, and apparently the risk for fatal cardiovascular events is higher among patients with more advanced liver fibrosis (type F3 or F4) [22,23]. A causal relationship between NAFLD and CVD will no longer be underappreciated by non-hepatologists or cardiologists as the body of scientific discoveries supports this link. A summary of the pathophysiological mechanisms contributing to the development of NAFLD and the associated increased risk for CVD is available below in Figure 1.

Risk factors for NAFLD are quite diverse. Beginning with alterations of hepatic microvasculature, increased arterial stiffness, and distortion of sinusoidal patterns which leads to intrahepatic endothelial dysfunction (increased levels of endocan, asymmetric dimethyl arginine, reduced circulation of endothelial progenitor cells) and activation of angiogenesis and vascular remodeling via VEGF, we can better understand the complex pathophysiological mechanisms behind an otherwise benign affliction [24]. Systemic inflammation expressed by circulating cytokines and chemokines released by the damaged liver (IL6, IL-1β, TNFα, sICAM-1, IL10/IL 17 ratio, and CXCL10) constitutes the backbone of an ongoing silent injury [24,25]. Altered homocysteine metabolism with increased oxidative stress within the liver, impaired nitric oxide formation, increased levels of oxidized low-density lipoprotein, and large very low-density lipoprotein have also been demonstrated in various studies [24,25,26]. Impaired hemostasis (as suggested by the increased levels of prothrombotic factors), hypercoagulability linked to atherosclerosis, and hepatic fat content via factors VIII, IX, XI, XII, and plasminogen inhibitor activators are mutual disruptions found in patients with both liver disease and CVD. Sharing the same intricate and complex process, CVD is generated when endothelial dysfunction (impaired redox status, increased levels of homocysteine, platelet activation, and systemic inflammation) is confirmed [25,26]. Atherosclerotic plaque formation and plaque instability emerge when the tonus of the vessel wall is altered and there is a release of pro-atherogenic factors including protein C levels, fibrinogen, and plasminogen activator inhibitor-1 together with increased hepatic insulin resistance, fatty acid accumulation, and increased levels of homocysteine adding to the injury [25].

Furthermore, mitochondrial malfunction and disruption is one of the main mechanisms of liver and heart damage. This should not come as a surprise since mitochondria are crucial organelles involved in cellular metabolism and homeostasis of energy [27]. Many elements and circumstances, including genetic predisposition, the presence of obesity and metabolic diseases, viral infections, and medications themselves, might have an impact on drug-induced mitochondrial dysfunction [27,28]. The heart has the largest concentration of mitochondria of any tissue and is the body’s most metabolically active organ [28]. Currently, mitochondrial dysfunction seems to be an important target for therapy to directly improve cardiac function. Alterations to ion homeostasis, shifting metabolic substrate usage, increased reactive oxygen species production, and decreased mitochondrial electron transport chain activity are all examples of mitochondrial abnormalities [27,28]. While the liver is a resilient organ, drug hepatotoxicity is a pervasive clinical issue that can have far-reaching effects on patient safety, the drug development process, and the healthcare system, as seen by increased morbidity and mortality [27]. To a molecular extent, some processes such as lipid peroxidation, mitochondrial depolarization and loss of lysosomal membrane integrity have been observed among patients treated with certain medications, as reported by Fard et al. in an experimental study on freshly isolated rat hepatocytes [29]. Cell injury is mediated by oxidative stress, and hepatocyte lysosomes and mitochondria play an important role in cell damage and death [29]. Chronic use of antidepressants, antimigraine medication, and other drugs that promote liver damage are used vivaciously among patients struggling with body dysmorphia, obesity, and anxiety disorders [27,28,29]. Clinicians should be aware of the potential of accelerating liver and even heart injury by altering mitochondrial homeostasis and to use caution when assessing patients with chronic treatments, as they can add to the injury. 

There are multiple intricate underlying mechanisms by which NAFLD increases the risk of CVD [24]. NAFLD is a progressive disease, and more severe NAFLD is associated with a significant inflammatory response and IR with a poor cardiometabolic response [25]. Therefore, a correct diagnosis and assessment of disease severity are of paramount importance due to the different metabolic consequences which correlate to different disease stages. 

Systemic inflammation, endothelial dysfunction, plaque instability, IR, oxidative stress, and altered lipid metabolism are some of the mechanisms involved in the complex pathogenesis of NAFLD-CVD [26]. The liver is a key metabolic organ that has a dual role of being both a target and a regulator of systemic inflammation. Deficient circulation and lipid uptake, increased hepatic lipogenesis, inadequate fatty acid oxidation, and altered export of lipids are found in NAFLD, which causes fat storage in the liver [27].

Visceral adipose fat appears to have a strong independent positive correlation with liver fat. However, the mechanism linking visceral fat to CVD is related to IR, which is also associated with an increased CV risk and atherosclerosis [30]. Besides fat accumulation in the liver, cardiac fat accumulation is an understated phenomenon [31]. In particular, the epicardial adipose tissue has unique properties. It is near the myocardium and adventitial layer and shares the same microcirculation, making it an ideal structure to exercise paracrine and vasocrine effect on the heart and blood vessels [32]. Unfortunately, the cardioprotective phenotype of the pericardium (suggested by the production of adiponectin) decreases substantially and gradually develops into a proinflammatory phenotype with the production of inflammatory markers and cytokines (IL 1β, IL-6, resistin, and TNFα) and access profibrotic pathways [33]. This enables the development of arterial structural changes, myocardial fibrotic tissue, and ventricular fibrosis, setting the foundation for coronary heart disease and heart failure [34]. 

Apolipoprotein-B-containing lipoproteins operate as damage-associated molecular patterns (DAMPs) that activate Toll-like receptors (TLRs) after being transported and oxidized within the subendothelial vascular wall [35]. Triglyceride- and apolipoprotein-3-containing lipoproteins further activate the NLRP3 inflammasome, which in turn controls pro-inflammatory marker and C-reactive protein (CRP) activity, encouraging vascular inflammation and atherosclerotic CVD. High-sensitivity CRP (hs-CRP), fibrinogen, and plasminogen activator 1 are also produced by the hepatic tissue and activate the coagulation process, exposing the body to atherothrombotic events [22,36]. We are constantly searching for non-invasive markers to assess risk or severity, and hs-CRP has been linked as an independent predictor of CV events in several large studies [37]. Kumar et al. reported that hs-CRP levels may be used as a surrogate marker for disease severity in a cohort of Indian patients with NAFLD [38]. In addition, the levels of prothrombotic factors, including fibrinogen, are increased in patients with NAFLD, and they directly correlate with underlying histology and severity of the disease, as reported by Potze et al. [39]. Therefore, subtle changes in inflammatory markers should be promptly observed. In addition to the occurrence of a systemic inflammatory response and also a pro-atherogenic basis via complex and intricate pathways, other metabolic imbalances have already been linked to disease development. Altered glucose metabolism and hepatic IR are metabolic catalysts of NAFLD and are crucial in both NAFLD and CVD pathogenesis [40]. The explanation is multifactorial and includes visceral obesity, elevated BMI, and, more importantly, ectopic fat distribution leading to IR, which is accompanied by compensatory persistent hyperinsulinemia. IR and compensatory hyperinsulinemia are associated with excessive hepatic glucose production in NAFLD [41]. These persistently abnormal biochemical responses promote atherosclerosis and plaque vulnerability.

As previously reported, vascular abnormalities and endothelial dysfunction are recognized as initial steps in disease progression in both CLD and CHD. Endothelial dysfunction is a complex process and is the foundation for atherosclerosis before plaque inflammation [42,43]. In addition, increased carotid intima-media thickness and calcification of arteries were also identified as precursors to more severe structural heart damage [44]. Furthermore, before hepatic inflammation and fibrosis, the hepatic microvasculature exhibits a distortion of the sinusoidal pattern and constriction of sinusoids by ballooned hepatocytes [45]. Impaired endothelial function is sometimes recognized as an early step in the development of atherosclerosis before the onset of fatty streaks or plaque inflammation [46]. Reduced availability of the vascular-protective vasodilatory molecule nitric oxide is a hallmark of endothelial damage, which is connected to superoxide oxidative stress, lipoprotein-mediated vascular inflammation, and IR. Asymmetric dimethyl arginine (ADMA), an antagonist of nitric oxide synthase (NOS), builds up in the liver in NAFLD due to a decrease in its ability to break it down. This causes a decrease in the availability of NOS as well as an accumulation of ADMA. Increased levels of homocysteine are also key factors for inducing oxidative stress and reducing the level of NO [47,48]. 

IR is another key metabolic feature of both cardiovascular and hepatic injury. Patients who have IR have a suppression of insulin’s effects, which causes the liver to continuously produce glucose [49]. Adipose tissue and muscles are the primary sites of peripheral IR, which disrupts insulin-mediated glucose uptake and utilization and causes an increase in adipose tissue breakdown. Postprandial hyperglycemia is caused by a deficiency in glycogen synthesis and glucose uptake in skeletal muscle [50]. As a result, the liver’s ability to absorb glucose rises. This causes the central metabolic coordinator carbohydrate response element-binding protein to activate, stimulating intracellular glycolysis and promoting ectopic de novo lipogenesis. The inhibition of lipolysis in adipose tissue is hampered by IR [51]. Triglycerides are hydrolyzed as a result of uncontrolled lipolysis, which releases significant amounts of free fatty acids (FFAs) and glycerol. The principal substrates for hepatic triglyceride production are these circulating FFAs, which build up in the liver and eventually cause intrahepatic triglyceride accumulation or steatosis. Selective hepatic IR boosts gluconeogenesis and lipogenesis in the liver [52]. Non-esterified FFAs released in excess into the bloodstream causes excessive absorption by tissues such as the liver, heart, pancreas, muscle, and endothelial cells, resulting in severe organ dysfunction or lipotoxicity, which further encourages peripheral IR, hepatic gluconeogenesis, hyperglycemia, and pancreatic beta-cell failure [53]. 

The genetic profile also contributes to disease outcome. Changes in the polymorphism of patatin-like phospholipase domain-containing 3 and transmembrane 6 superfamily member 2 are linked to an increased risk of liver steatosis, fibrosis, and cancer [31,54]. However, a fascinating finding from recent research is that those who carry the identified PNPLA3 and TM6SF2 genetic variations are likely to exhibit a cardioprotective phenotype [31,55]. Therefore, this is a field that is worth exploring, as it can be used for risk assessment and risk stratification. In addition to genetic changes, dysbiosis has been identified as a risk factor for the emergence of cardiometabolic illnesses such atherosclerosis, hypertension, T2DM, and NAFLD [56,57]. Although certain bacteria abundance patterns have been regularly replicated in various disorders, the results of the heterogeneous studies did not consistently demonstrate a gut microbiota profile that is unique to NAFLD [58].

## 3. Cardiovascular Morbidity and Mortality and NAFLD

While some cohort studies revealed that NAFLD patients may have increased cardiovascular mortality, other research was unable to support this idea. For instance, a recent meta-analysis by Targher et al. revealed that people with NAFLD had a 64% higher likelihood than those without NAFLD of having fatal or nonfatal cardiovascular events. Non-fatal events included myocardial infarctions requiring coronary revascularization, strokes, and angina pectoris. The findings of Wu et al. differ from those of Tagher et al. in that they did not find a clear connection between the presence of NAFLD and an increase in cardiovascular mortality [59].

A distinct risk group can be identified in patients with NAFLD who also have concurrent T2DM, NASH, or advanced fibrosis (F3–F4) [60]. NAFLD prevalence in T2DM patients ranges from 70% to 95%; in those with severe obesity, it can reach up to 98% [61,62]. NAFLD is linked to higher all-cause mortality, but there seems to be a stage-dependent relationship between it and CVD mortality [63]. Because NAFLD increases CV events and mortality even in the absence of metabolic comorbidities such as diabetes, dyslipidemia, and hypertension, as well as their consequences and precursors [20,63], these conditions are often present in people with NAFLD. Additionally, hepatic fibrosis is a significant predictor of liver-related outcomes and is linked to death from all causes, CV mortality, cirrhosis, hepatocarcinoma, and infectious illnesses in NAFLD patients [23,63,64]. Diabetes has a significant impact on all-cause and CV mortality in NAFLD patients [63,64]. Therefore, it is important to properly characterize the level of fibrosis following the diagnosis of NAFLD [64,65]. There is proof that patients with T2DM who have NAFLD have higher risk of chronic renal disease, macrovascular damage, and diabetic microvascular complications [65,66]. Similar results were published by Zhou et al. in a systematic review and meta-analysis, indicating that NAFLD raises the risk of CVD in populations with comparable diabetic profiles. An early assessment of cardiovascular risk for diabetic patients with NAFLD may reduce CVD morbidity and mortality [67].

Hs-CRP is a non-specific and sensitive marker for detecting inflammation that was proposed for its potential to add predictive value in several chronic diseases [68]. A follow-up study with a median follow-up period of 22.3 years of MAFLD patients performed by Huang et al. supported the role and valuable contribution of hs-CRP as an independent predictive factor of poor prognosis in patients with MAFLD. According to the findings of a Cox regression analysis, hs-CRP levels below 0.5 mg/dL are an independent risk factor for mortality from all causes, cardiovascular disease, and malignancy. Hs-CRP could successfully differentiate MAFLD with poor prognosis and mortality risk at a level of 0.5 mg/dL [69,70]. Higher fibrosis grades were seen in patients with abnormal angiography according to a cross-sectional comparative investigation of 103 consecutive NAFLD patients by Nayebi et al., which revealed a significant difference in the level of hs-CRP according to elastography fibrosis grade [71].

The prevalence of arterial hypertension in NAFLD patients ranges from 40 to 70%, and recent research has demonstrated that NAFLD is closely linked to an elevated risk of incident prehypertension and hypertension [72]. People with hepatic steatosis on ultrasonography had significantly higher mean blood pressure readings than people without NAFLD according to the Finland OPERA Study’s examination of patients’ 24 h blood pressure [73]. Another meta-analysis by Li et al. reported that hypertension was strongly linked to a higher probability of incidentally developing NAFLD. In contrast, nine studies that included information on 46,487 participants examined the impact of NAFLD on incident hypertension. A combined analysis revealed a substantial correlation between NAFLD and a higher incidence of hypertension [19].

Patients with NAFLD are more likely to develop both clinical and subclinical atherosclerosis. Patients with NAFLD may also be more susceptible to the development of atherosclerotic CVD due to an imbalance in procoagulant factors [74]. In addition to elevated levels of fibrinogen and von Willebrand factor and lower levels of antithrombin III and protein C, they usually exhibit elevated serum concentrations of the factors FVIII, FIX, FXI, and FXII [75]. Another element that may influence atherogenesis and plaque instability in NAFLD patients is altered vascular endothelial growth factor blood concentrations. The presence of active angiogenesis and higher serum levels of vascular endothelial growth factor in NAFLD patients indicate vascular remodeling, which is linked to both plaque production and plaque instability [76]. In a recent meta-analysis published by Tang et al., out of 7951 NAFLD patients, more than one-third had carotid atherosclerosis, with an OR of 3.20 and a stroke prevalence of 5.04%. While the incidence of hemorrhagic stroke was found to be 2.22%, the incidence of ischemic stroke in NAFLD was found to be 6.05%. Except for an elevated level of alanine aminotransferase, there was no significant factor influencing the presence of stroke in NAFLD, highlighting the need for routine assessment of carotid atherosclerosis in NAFLD [77]. Arai et al. reported that high-risk CIMT is strongly and independently correlated with advanced fibrosis. In patients with NAFLD, non-invasive fibrosis markers and scoring systems may be used to evaluate the risk of atherosclerosis progression. Moreover, the degree of fibrosis was found to significantly correlate with max-CIMT but not with steatosis, inflammation, or ballooning. Max-CIMT 1.2 mm was independently associated with advanced fibrosis, male gender, older age, and hypertension. In comparison to the non-advanced fibrosis group, the advanced fibrosis group had a considerably greater prevalence of max-CIMT 1.2 mm [74]. A similar result was published by Jin et al., and the authors reported that liver disease fibrosis scores were significantly associated with coronary severity, vascular calcification, and cardiovascular events [78]. Even after adjusting for covariates associated with CVD risk factors in a significant number of studies, NAFLD remains an underestimated and independent risk factor for atherosclerotic CVD [24,31]. In addition, cardiac arrhythmias are exceedingly prevalent in individuals with heart disease and can have catastrophic implications, such as an increased risk of heart failure, stroke, and sudden death. Atrial fibrillation is the most prevalent sustained form of heart arrhythmia [59,64]. Interestingly, however, cardiologists have not completely acknowledged NAFLD as a major risk factor for CVD, and current guidelines do not highlight NAFLD as a key intervention target for CVD, DM, or dyslipidemia [64,79]. Emerging lines of evidence also suggest that people with NAFLD have an elevated risk of cardiac arrhythmias such as atrial fibrillation and ventricular arrhythmias [59,79]. QTc interval prolongation is a well-known risk factor for ventricular arrhythmia and premature cardiac death. Preliminary evidence has connected NAFLD to QTc interval prolongation in individuals with T2DM and the general population [79]. In 2014, a cross-sectional study of 400 outpatients with T2DM first reported that the presence of ultrasound-diagnosed NAFLD was significantly associated with an increased QTc interval [80]. Similar results were reported by Hung et al. in a cross-sectional study. The authors found that mild, moderate, and severe NAFLD detected by ultrasonography was associated with higher risks for QTc prolongation [81].

Patients with NAFLD/NASH are more likely to have subclinical CVD and several other cardiovascular risk factors [46]. A summary of the current cardiovascular risk scores is available in Table 1.

## 4. Tools and Risk Scores for Assessing Cardiovascular Events in NAFLD: An Unmet Clinical Need

Because of the inherent limitations of using risk scores based on conventional CV risk-factor-derived multivariable statistical models to identify at-risk patients, the current global risk prediction studies are imperfect even though they may contribute to describing, at least in part, the relationship between NAFLD and increased CV risk [24,31]. Additionally, we are aware that these risk assessment models frequently do not take into consideration a number of the important CV disease risk factors, such as IR, obesity, and increased triglycerides. It has already been argued that the Framingham risk score understates the risk of CV disease in this population due to the similarities between metabolic syndrome and NAFLD [69,70]. As a result, it may not be appropriate to risk-stratify individuals with NAFLD solely based on the most recent CV risk score systems. More research is needed to create straightforward, inexpensive robust biomarkers (or algorithm-based scores) of NAFLD status, including its immediate cardiometabolic effects, before we can evaluate its improved discriminant value when applied to current CV risk-prediction models in cohort studies [78].

With varying degrees of severity in each stage of simple steatosis and NASH, NAFLD progresses differently (often over several years). Except for more severe cases of NASH+ fibrosis, these stages are typically reversible [15,16,19]. Cardiovascular risk is increased as NAFLD becomes more severe. Even though ultrasonic imaging can only detect steatosis when 30% of the liver is damaged, it is still advised as the first line of inquiry to confirm the existence of fatty liver due to its broad availability and inexpensive cost [79]. 

To diagnose the NAFLD stage and the degree of NASH and fibrosis by histological examination, as well as to track the course of the illness, liver biopsy is currently the “gold standard” (accounting for possible errors caused by sampling variability) [80]. Currently, the non-invasive approach is used in clinical practice. Several widely used tests are available with different degrees of accuracy to assess the presence of liver steatosis and fibrosis (Table 2). Hepatic steatosis is the mildest type of MAFLD/NAFLD, with a lipid content in the hepatic parenchyma of more than 5% and no portal or lobular inflammation [69,70,81]. In their lifetimes, approximately 4% of people with liver steatosis are anticipated to develop fibrosis [15,69,70].

NAFLD can develop in individuals who are not overweight. In recent years, emerging evidence has demonstrated that lean NAFLD is a progressive condition and that predominantly male patients with lean NAFLD exhibited some features of MetS and might have worse outcomes than obese-NAFLD [82]. Whether lean-NAFLD has lower mortality and lower incidence of cirrhosis, malignancy, CVD, or metabolic comorbidities compared to overweight/obese NAFLD remains a matter of debate [15,16]. However, more recently, a higher incidence of CV events and higher mortality rates among patients with lean NAFLD compared to obese patients has been reported. More specifically, a recent paper published by Wijarnpreecha et al. in a cross-sectional and longitudinal analysis of more than 18,000 patients reported that lean patients with NAFLD had higher mortality despite lower incidence of cirrhosis and diabetes and similar incidence of CVD and cancer [83]. This specific category of patients requires even more attention during follow-up compared to non-lean patients with NAFLD. Moreover, a comparative analysis performed by Kim et al. among patients without NAFLD, with obese NAFLD, and with lean NAFLD reported alarming results: subjects with lean NAFLD had a significantly higher ASCVD score and prevalence of a high ASCVD risk than those with obese NAFLD and without NAFLD. Additionally, lean subjects with significant liver fibrosis had a higher probability of ASCVD than obese subjects in the subpopulation with NAFLD [25]. Indeed, the classical phenotype of NAFLD patients occur in obese or overweight individuals. To date, the long-term outcomes of lean-NAFLD and MetS are still not fully understood, and more long-term studies are required to fill in the research gap.

The NAFLD Liver Fat Score has its origins in a Finnish population [75]. Insulin levels and the degree of hepatic steatosis are connected because IR is a key risk factor for the onset of MAFLD/NAFLD. Additionally, AST is released from damaged hepatocytes, and an AST level rise indicates liver disease [70]. Additionally, it has demonstrated a positive correlation with CVD incidence and death, outcomes that are closely linked to metabolic syndrome and T2DM [56,61].

The fatty liver index is based on BMI, the AST/ALT ratio, and the existence of T2DM, and this measure evaluates MAFLD/NAFLD [84,85,86,87]. Similarly, the risk of premature death and the occurrence of MAFLD/NAFLD were positively and virtually linearly linked with both enzymes. Additionally, this score shows a strong connection with steatosis grades identified by ultrasonography, but it has not been verified for NASH up to this point [88]. The fatty liver index and incident CVD have a strong correlation, as reported by Zou et al. The fatty liver index also signals the baseline and potential future development of CVD on long-term follow-up across weight categories and the fatty liver index spectrum [89]. Clinicians and other interested parties can use these data to help manage and prevent CVD. Patients with high fatty liver index levels need to be informed about their elevated risk of cardiovascular disease in the future.

The APRI (AST-to-Platelet Ratio Index) is another easy-to-use tool to quickly assess the presence of fibrosis. It has been validated for the identification of NAFLD and can identify advanced fibrosis in those with viral infection. APRI is regarded as a good predictor of progressive fibrosis in patients with NAFLD [90]. Nevertheless, several authors have advocated against its broad use, mostly due to its poor accuracy in fibrosis staging. Numerous papers have studied the use of APRI scores for assessing CV risk in metabolic patients [91]. APRI has previously been proven to be a reliable score for predicting liver fibrosis in people with viral hepatitis, with a cut-off of 0.5 for fibrosis and 1.5 for cirrhosis. The research by De Matteis et al. demonstrated that APRI significantly correlates with CV risk and, when raised, defines a significant rise in CV risk for both genders, particularly for females. When APRI is elevated, an increase in CV risk is seen. This elevation of risk is somewhat high in patients aged 51 to 65, but it is significantly greater in younger, premenopausal women [43]. The value of APRI is a trustworthy and simple-to-use score for assessing CV risk in metabolic patients.

Another widely used score is the NAFLD fibrosis score. Age, glucose levels, BMI, albumin and platelet count, and AST/ALT ratio are the parameters used. This score is widely used to predict advanced fibrosis. Particularly, patients with NAFLD have reduced levels of albumin and albumin-binding function. A high score (>0.68) significantly increased the probability of death in MAFLD/NAFLD patients by four times [92]. Even though this score accurately predicts morbidity and mortality at all stages of fibrosis, it has limited relevance in predicting changes in fibrosis [93].

In addition to risk-stratifying methods based on fibrosis for the MAFLD/NAFLD spectrum, many authors have proposed non-invasive scoring systems as screening tools for liver disease. However, some healthcare experts continue to emphasize liver biopsy as the gold standard for diagnosis while arguing for a thorough characterization of biopsy indications. However, fatty liver index, APRI, fibrosis-4 index, and NAFLD fibrosis score, in contrast to other available risk scores, depend on commonly requested parameters, making them easier to utilize in daily clinical practice [93]. Additionally, patients who have more metabolic anomalies tend to produce higher scores, making these scores more accurate as the patient’s condition deteriorates.

## 5. The Future of NAFLD: Emerging Therapeutic Approaches

Although NAFLD is a common disease, there are no approved therapies. As previously reported, the leading causes of death in patients with NAFLD are complications of cardiometabolic disease. Therefore, efforts are directed toward developing drugs that target both NAFLD and cardiometabolic risk factors. Currently, several therapeutic agents have been proposed and are under assessment [94]. Given the bidirectional relationship between T2DM and insulin resistance and hepatic lipid accumulation, numerous anti-diabetic drugs have been tested as NASH therapeutics in experimental models during clinical trials [94,95]. For now, modulators of glucagon-like peptide 1 (GLP1) activity, insulin-sensitizing thiazolidinediones, and inhibitors of the sodium–glucose cotransporter 2 (SGLT2) have been tested in clinical trials with promising results. GLP1R agonists reduce hepatic steatosis and markers of liver damage as reported by Armstrong et al. [95]. Liraglutide and semaglutide have shown efficacy in reducing hepatic lipid content as well as levels of liver enzymes and inflammatory status. The inhibition of dipeptidyl peptidase 4 has also been targeted therapeutically to enhance GLP1 activity, and linagliptin, saxagliptin, and sitagliptin are currently involved in clinical trials [94,95,96]. Thiazolidinedione insulin sensitizers such as pioglitazone and rosiglitazone are used to treat T2DM by improving peripheral insulin sensitivity. Inhibitors of SGLT2 (canagliflozin, dapagliflozin, and ertugliflozin) are another category of agents used to treat diabetic patients by reducing glucose reabsorption [94,95]. Farnesoid X receptor agonists, bile acids, and synthetic bile acids are also under study. Among them, obeticholic acid, a bile acid derivative that activates the farnesoid X receptor displayed optimistic results in a study by Mudaliar et al.; it increased insulin sensitivity and reduced markers of liver fibrosis and inflammation in patients with NAFLD andT2DM [97]. The PAAR family, including elafibranor, acts by reducing hepatic steatosis, inflammation, and fibrosis in NAFLD. The thyroid hormone receptor, stearoyl-CoA desaturase 1 (SCD1), a fatty acid desaturase, ketohexokinase inhibitors, fibroblast growth factors, FGF21 analogs, and mitochondrial pyruvate carrier inhibitors are currently under study [94,95,96,97]. The future is looking bright, as these metabolic-targeted therapies may yield positive results in managing NAFLD and T2DM patients while reducing additional cardiometabolic risk factors and, by doing so, reducing mortality rates.

## 6. Conclusions

The causal bidirectional relationship between CVD and NAFLD is more tangible nowadays. Early clinical intervention in at risk patients could alter the disease course. Moreover, patients who share multiple cardiometabolic risk factors who are at risk for developing CV events should benefit from early clinical and therapeutic intervention. Current guidelines do not recognize NAFLD as an independent risk factor for CVD, despite recent research indicating NAFLD’s involvement in incident CVD. Improving patient safety requires a greater understanding of the biopsy-free scores that are available for staging. Both NAFLD and CVD are silent pandemics, which are constantly expanding, and they compel clinicians to look for alternate methods of screening, early detection, and follow-up. Prospective long-term studies with homogeneous diagnostic criteria, considering not only the presence but also the severity of NALFD, are necessary to test if this diagnosis can improve cardiovascular disease risk stratification. New and inclusive risk scores are needed to address complex patients who are at risk of developing either CV events or progression toward more advanced and irreversible liver disease with overall poor outcome. Emerging new therapies could change the face of NAFLD patients.

## Figures and Tables

**Table 1:** Cardiovascular risk scores.

Risk Score	Risk Predictors	Clinical Use and Benefits
CT Coronary Artery Calcium score (CAC)	Degree of coronary artery calcification (absent, discrete, moderate, accentuated)	Independent added value in the prediction of all-cause mortality and mortality owing to coronary disease in asymptomatic persons.
Systematic Coronary Risk Estimation 2 (SCORE2)	Age, smoking status, systolic blood pressure, total cholesterol, and HDL cholesterol	✓ It calculates a person’s 10-year chance of suffering a fatal or non-fatal CVD event (myocardial infarction or stroke) in the age range of 40 to 69 in apparently healthy individuals with risk factors that are untreated or that have been stable for several years. ✓ Excludes those with known cardiovascular disease (CVD), other high-risk illnesses such as diabetes mellitus (DM), other uncommon hereditary lipid or blood-pressure problems, and pregnant women.
Systematic Coronary Risk Estimation 2-Older Persons (SCORE-OP)	Age (per year), history of diabetes, current smoking, SBP (per 10 mmHg), total cholesterol, and HDL cholesterol	SCORE2-OP to estimate 5- and 10-year risk of incident CVD in older individuals aged over 70 years without pre-existing CVD.
QRISK2	Age, gender, total cholesterol/high-density lipoprotein ratio, systolic blood pressure, smoking status, diabetes mellitus, family history, treated hypertension, BMI, Townsend deprivation score, self-assigned ethnicity, rheumatoid arthritis, atrial fibrillation, and chronic renal disease	✓ Decisions regarding a patient’s therapy, such as lifestyle recommendations or pharmacological treatment, can be based on their 10-year risk of CVD. ✓ The percentage of persons who are evaluated for secondary reasons before receiving statin medication and have a 10-year CVD risk of 10% or higher. ✓ Adults 85 years of age and older, as well as those with pre-existing cardiovascular disease (CVD), type 1 diabetes, chronic renal disease, or familial hypercholesterolemia, should be viewed as being at a higher risk of CVD events without using QRISK2.
REYNOLDS Risk Score (RRS)	Age, gender, systolic blood pressure, total cholesterol, high-density lipoprotein, smoking status, hemoglobin A1c (women only), family history, and high-sensitivity C-reactive protein	✓ is regarded as a good screening tool for CVD risk. ✓ RRS predicts the onset and progression of subclinical atherosclerosis ✓ has been shown to improve CVD risk prediction in both females and males
Framingham Risk Score (FRS)	Age, gender, systolic blood pressure, total cholesterol, high-density lipoprotein, smoking, diabetes mellitus, and blood pressure medications	✓ the most widely used and applicable method for predicting the person’s chance of developing CVD in long term ✓ the 10-year risk score can be derived as a percentage, which can then be used to inform the decision about initiating lipid-lowering therapy for primary prevention
Atherosclerosis Cardiovascular Disease (ASCVD) risk score	Age, sex, race, total cholesterol, HDL cholesterol, systolic blood pressure, blood-pressure-lowering medication use, diabetes status, and smoking status	The 10-year ASCVD risk score calculation may be the first step in implementing preventive measures such as lipid profile and blood pressure management.
European Society of Cardiology-Systematic COronary Risk Evaluation (ESC-SCORE)	Age, systolic blood pressure, total cholesterol, and total cholesterol/high-density lipoprotein ratio	Individual risk of the 10-year risk for a fatal CV event.
Prospective Cardiovascular Munster Study (PROCAM) risk score	Age, LDL cholesterol, smoking, HDL cholesterol, systolic blood pressure, family history of premature myocardial infarction, diabetes mellitus, and triglycerides	✓ A simple scoring system for determining the risk of acute coronary events based on the PROCAM study’s 10-year follow-up. ✓ The PROCAM coronary risk score emphasizes the importance of treating atherogenic dyslipidemia, which includes low HDL cholesterol and high triglycerides, as a factor in higher risk. ✓ Additionally, the PROCAM cerebral ischemia risk score fills a void for a straightforward, widely applicable risk score that enables clinicians to target the right multifactorial strategy to lower stroke risk.

**Table 2:** Biopsy-free scoring systems available for assessing the stages of steatosis and fibrosis.

Biopsy-Free Scoring Systems and Clinical Use	Name of Score/Biomarker	Algorithm/Formula Used	Cut-Off
Assessment of hepatic steatosis used in both MAFLD and NAFLD	NAFLD ridge score	−0.614 + 0.007 × ALT−0.214 × HDL C + 0.053 × triglyceride + 0.144 × HbA1c + 0.032 × WBC + 0.132 × Hypertension	0.24 and 0.44
	NAFLD liver fat score	1.18 × MS + T2DM (2 if yes; 0 if no) + 0.15 × fasting insulin (mU/L) + 0.04 × AST(U/L) 0.94 × (AST/ALT) 2.89.	>−0.640 predictable for NAFLD
	Hepatic steatosis index	8 × ALT/AST + BMI (+2 if type 2 diabetes yes, + 2 if female)	30 and 36
	Fatty liver index	e y/(1 + ey) × 100 y = 0.953 × ln(triglycerides, mg/dL) + 0.139 × BMI, kg/m2 + 0.718 × ln (GGT, U/L) + 0.053 × waist circumference, cm—15.745)	30 and 60
Assessment of fibrosis	APRI	(AST(IU/L)/AST(ULN))/platelet count (10^9/L)) × 100	0.5 and 1.5
	Fibrosis-4 index	age × AST (IU/L)/platelets (× 109/L) × √ ALT (IU/L)	low risk, FIB-4 < 1.3; intermediate risk, FIB4 1.3–2.66; high risk, FIB-4 ≥ 2.67.
	BARD score	(BMI > 28 = 1 point) + (AAR > 0.8 = 2 points) + (DM = 1 point).	A BARD score of 2–4 points was associated with F3 or F4 stages of fibrosis
	NAFLD fibrosis score	−1.675 + (0.037 × age [years]) + (0.094 × BMI [kg/m2 ]) + (1.13 × IFG/diabetes [yes = 1, no = 0]) + (0.99 × AST/ALT ratio)—(0.013 × platelet count [×109 /L])—(0.66 × albumin [g/dL])	−1.455 and 0.675 scores > 0.675 suggest a high risk of fibrosis
	Enhanced liver fibrosis test	2.494 + 0.846 ln(HA) + 0.735 ln(PIIINP) + 0.391 ln(TIMP1).	7.7: exclusion of fibrosis, 9.8: identification of fibrosis
	FibroMax	A non-traumatic diagnostic method and a unique alternative to liver biopsy.	0–0.31 F0–F1 0.32–0.0.58 F2 0.59–1.00 F3–F4
Additional non-invasive scores	BAAT score	(BMI ≥ 28 = 1 point) + (age ≥ 50 years = 1 point) + (ALT ≥ 2N (1 point)) + (triglycerides ≥ 1.7 mmol/L (1 point)).	assess risk of fibrosis in overweight patients with NAFLD, score of 0 had 100% negative predictive value for diagnosis of septal fibrosis
